# Therapeutic potential of extracellular vesicles in preclinical stroke models: a systematic review and meta-analysis

**DOI:** 10.1136/bmjos-2019-100047

**Published:** 2020-02-24

**Authors:** Josephine M Thomas, Catriona J Cunningham, Catherine B Lawrence, Emmanuel Pinteaux, Stuart M Allan

**Affiliations:** Division of Neuroscience and Experimental Psychology and Lydia Becker Institute of Immunology and Inflammation, Faculty of Biology, Medicine and Health, Manchester Academic Health Science Centre, The University of Manchester, Manchester, UK

**Keywords:** stroke, extracellular vesicles, preclinical

## Abstract

**Objectives:**

Currently there is a paucity of clinically available regenerative therapies for stroke. Extracellular vesicles (EV) have been investigated for their potential as modulators of regeneration in the poststroke brain. This systematic review and meta-analysis aims to provide a summary of the efficacy of therapeutic EVs in preclinical stroke models, to inform future research in this emerging field.

**Methods:**

Studies were identified by a comprehensive literature search of two online sources and subsequent screening. Studies using lesion volume or neurological score as outcome measures were included. Standardised mean difference (SMD) and 95% CIs were calculated using a restricted maximum likelihood random effects model. Publication bias was assessed with Egger’s regression and presented as funnel plots with trim and fill analysis. Subgroup analysis was performed to assess the effects of different study variables. Study quality and risk of bias were assessed using the CAMARADES checklist.

**Results:**

A total of 20 publications were included in the systematic review, of which 19 were assessed in the meta-analysis (43 comparisons). Overall, EV interventions improved lesion volume (SMD: −1.95, 95% CI −2.72 to 1.18) and neurological scores (SMD: −1.26, 95% CI −1.64 to 0.87) compared with control groups. Funnel plots were asymmetrical suggesting publication bias, and trim and fill analysis predicted seven missing studies for lesion volume. Subgroup analysis suggested administration at 0–23 hours after stroke was the most effective timepoint for EV treatment. The median score on the CAMARADES checklist was 7 (IQR: 5–8).

**Conclusions:**

EVs may offer a promising new avenue for stroke therapies, as EV-based interventions had positive impacts on lesion volume and neurological score in preclinical stroke models.

**PROSPERO registration number:**

CRD42019134925.

**Strengths and limitations of this study**

- All data and codes will be freely available after publication and study protocol was registered prior to completion.
- We assessed publication bias and study quality as well as subgroup analyses to attempt to explain heterogeneity across included studies.
- Study numbers were low for some subgroups making meta-analysis challenging.
- Heterogeneity across extracellular vesicle sources was not accounted for.

## Introduction

Stroke is a global health problem responsible for 6.7 million deaths annually.^1^ In the UK alone there are 1.2 million stroke survivors, a third of whom live with resulting disabilities, including impaired motor function, impaired speech and language communication and cognitive decline.^2^ Stroke occurs when blood flow to the brain becomes disrupted, leading to hypoxia and subsequent cell death. This can be caused by a blockage of blood vessels (ischaemic stroke) or the rupturing of vessels (haemorrhagic stroke). Treatments for both types of stroke are extremely limited. Tissue plasminogen activator is the only Food and Drug Administration-approved agent for ischaemic stroke but its use is limited by its narrow window of administration of up to 4.5 hours from symptom onset. Thrombectomy using mechanical devices is also approved for some ischaemic stroke cases, though only an estimated 10% of patients are eligible for this treatment.^3^ With no biological agents approved for haemorrhagic stroke, surgery to repair damaged blood vessels is the only existing treatment. Currently the treatments available for both stroke subtypes are aimed at aiding reperfusion and thus reducing the damage caused by stroke. This highlights a huge unmet need for restorative and regenerative therapies to alleviate functional deficits and the long-term disabling effects of stroke.

One promising candidate for stroke therapy is mesenchymal stem cells (MSC). A recent systematic review and meta-analysis of 141 studies demonstrated that bone marrow-derived MSC transplantation leads to improvements in functional outcomes in preclinical models of cerebral ischaemia.^4^ Initially it was thought that MSCs promote repair and ameliorate functional deficits by differentiating into parenchymal cells.^5^ However, more recent studies have shown that MSCs do not engraft and differentiate in the stroke brain,^6^ but rather accumulate in the lungs following intravenous injection.^7^ Instead, the paracrine effects of the implanted MSCs are likely responsible for the restorative effects observed in previous studies. MSCs secrete an array of growth factors, extracellular vesicles (EV) and cytokines which together could mediate these restorative processes.

EVs are nanoscale membrane-bound vesicles which carry cargoes including DNA, RNA and proteins that are involved in intercellular signalling. EVs can be subcategorised according to their biogenesis. Exosomes are 30–100 nm in diameter and of endosomal origin while microvesicles (50–2000 nm) and apoptotic bodies (500–4000 nm) are formed by outward budding of the plasma membrane of the cell and apoptotic cell fragments, respectively.^8^ EVs are found in biological fluids, and have been shown to enter the circulation, indicating that their effects are not limited to the cells surrounding their origin. At their target sites, EVs release their cargo by fusion with either the plasma membrane or membranes of the endocytic components, allowing signalling molecules such as nucleic acids to alter gene expression in the recipient cell.^9^ Reported biodistribution of injected EVs varies between studies, and the accumulation in the liver, spleen and lymphatic system may be influenced by different routes of administration.^10^ Although much remains unknown about the biological actions of EVs, the field has grown rapidly in recent years, leading to the establishment of the International Society for Extracellular Vesicles (ISEV) in 2012. Importantly, ISEV released a set of guidelines for researchers to adhere to when studying EVs, in particular to aid characterisation of EVs and improve reporting of functional studies.^11^ EVs are not associated with the tumorigenic and immunogenic caveats of cell therapies, and treatments can be prepared and stored in advance, making EV-based therapies an attractive avenue for future medical research.

Since 2013, several groups worldwide have investigated the potential therapeutic benefits of EVs in preclinical stroke models, the results of which will be analysed in part in this review. A major issue for the treatment of stroke, among other diseases, is translation; many therapeutic agents effective in experimental stroke have failed to show efficacy in human trials. Dirnagl and Macleod^12^ highlight a number of reasons for this, including bias in preclinical studies and poor study quality. Currently there is no systematic review or meta-analysis examining the therapeutic potential of EVs in preclinical stroke models.

### Aims

The aim of this review and subsequent meta-analysis is to examine the potential efficacy of EVs in preclinical stroke models, and to assess the methodological quality of included studies to aid future research in this field and improve reporting.

## Methods

This systematic review was conducted in accordance with the Preferred Reporting Items for Systematic Reviews and Meta-Analyses statement,^13^ and the protocol was preregistered in the PROSPERO database.

### Search strategy

PubMed and Embase (OVID) were searched according to the search strategy outlined in the PROSPERO protocol. Articles published from January 2013 were assessed by two independent reviewers (CJC, JMT), and the reference lists from relevant reviews were also screened for additional articles. The last search was performed on 16 May 2019.

### Study selection & data extraction

Full texts of relevant articles were screened for studies investigating the therapeutic effects of EVs in preclinical stroke models. Studies were included if lesion volume and/or neurological scores of any scale were reported as outcome measures. Studies in which EVs were not administered, or those without appropriate controlled groups were excluded. Studies were excluded if full English language texts were not available. Qualitative data were extracted for subgroup analyses and narrative synthesis. This included information on the study design including methods of EV isolation, characterisation and details of the intervention including route and timepoints of administration.

For lesion volume and neurological score, mean values and SEM or SD were extracted as reported in the articles. Where raw values were not given in text, the online graphical tool WebPlotDigitizer (https://automeris.io/WebPlotDigitizer/) was used to extract data from figures. Estimates were cross-checked by a second reviewer and any conflicts >10% difference were resolved by discussion. In instances where the exact numbers of animals in each group were not reported and it was unclear whether error bars represented SEM or SD, the authors were emailed for clarification. If the data were not made available following two attempts to contact the authors, those studies were excluded from the meta-analysis.

Study quality was assessed using the CAMARADES (Collaborative Approach to Meta-analysis and Review of Animal Data in Experimental Studies) risk of bias checklist,^14^ with the following categories: (1) publication in peer-reviewed journal, (2) statement of control of temperature, (3) randomisation of treatment or control, (4) allocation concealment, (5) blinded assessment of outcome, (6) avoidance of anaesthetics with marked intrinsic properties, (7) use of animals with comorbidities, (8) sample size calculation, (9) statement of compliance with regulatory requirements, and (10) statement regarding possible conflict of interest. Studies were recorded as positive for allocation concealment if animals were randomised to treatment groups after surgery or if blinding to surgery groups was explicitly stated. Comorbidities included diabetic, hypertensive or aged animals. Data were extracted by two independent reviewers (CJC, JMT) and any disagreements were resolved through discussion with a third author (SA).

Studies were grouped by timepoint of EV administration with the following subgroups: administration before induction of stroke and up to reperfusion time; administration from reperfusion to 23 hours after stroke; 24–48 hours after stroke, and multiple administrations of EVs across these timepoints. Routes of EV administration were grouped into studies delivering EVs intravenously, intra-arterially and other routes. Studies were classified as including exosome characterisation if data from the characterisation were included in the article. Finally, studies were also grouped depending on whether investigators were blinded to treatment groups when measuring outcomes, if they included randomisation to intervention groups and if researchers were blinded to surgery, according to the CAMARADES checklist as described above.

### Statistical analysis

All statistical analyses were performed in RStudio V.1.2.1335 (RStudio, USA) using the metafor package (http://www.metafor-project.org/doku.php, RRID:SCR_003450). Standardised mean difference (SMD) effect sizes were calculated using Hedges’ *g*. A random effects meta-analysis was conducted using the restricted maximum likelihood method for both lesion volume and neurological scores. Heterogeneity was assessed using the I^2^ statistic. The random effects model was chosen due to the high heterogeneity in the data set. Subgroup analyses were performed to assess the effects of different study variables including randomisation, blinding and timepoint of administration of effect size. Independent random effects models were fitted to subgroups, and estimates compared with a Wald-type test. Publication bias was assessed using funnel plots and confirmed with Egger’s regression. Trim and fill analysis was used to determine the effect size when accounting for unpublished studies.

## Results

### Qualitative synthesis

**Figure 1:**
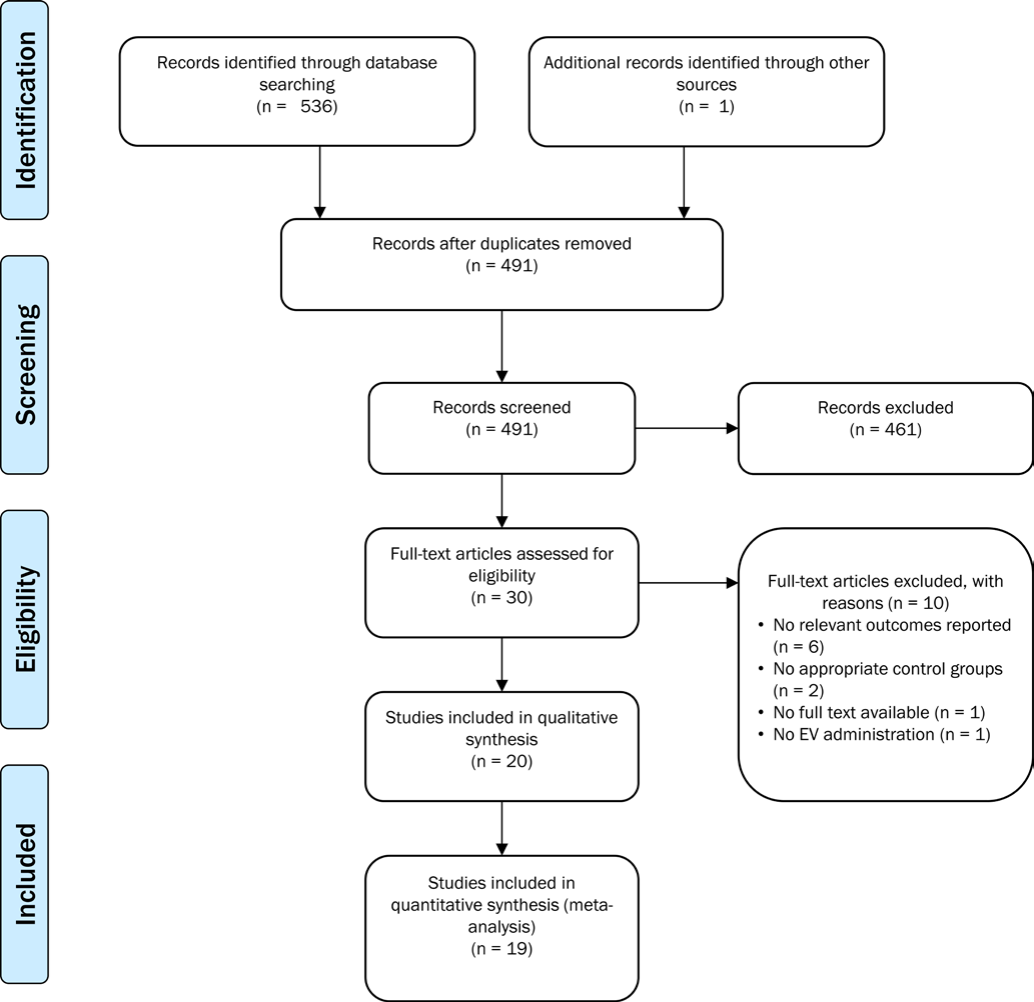
Flow diagram showing search strategy and inclusion and exclusion of studies for systematic review and meta-analysis. EV, extracellular vesicle.

A total of 491 articles were identified from our literature search and reference list screening (figure 1). Of these, 20 met the inclusion criteria for our systematic review and study details are summarised in table 1. Most of the studies used ischaemic stroke models (n=18), with only two investigating intracerebral haemorrhage. Rats (n=15) and mice (n=4) were the animals most commonly studied, and pigs (n=1) were also used. Studies were heterogeneous in the functional outcome measures assessed with 20 different behavioural tests and six neurological score scales used including the modified neurological severity score, Rogers, Belayev and Longa tests. A total of 19 studies including 525 animals (control n=209, treatment n=316) were included in the meta-analysis for a total of 43 comparisons (n=22 for lesion volume, n=21 for neurological score). One study was excluded from the meta-analysis as numbers of animals in experimental groups were not available.

**Table 1:** Study design data extracted from articles included in systematic review

Publication	Year	Stroke model	Species	EV therapy	Isolation method	Route of administration	Dose	Timing poststroke	Lesion volume (Y/N)	Functional outcomes	Study length
Chen *et al*^27^	2016	Transient distal MCAO, filament	Rat	Mini-pig AMSCs/AMSC-derived exosomes	Ultracentrifugation	Intravenous	100 µg	3 hours	Y	Corner test	60 days
Doeppner *et al*^28^	2015	Transient MCAO, filament	Mouse	Human BMSC-derived exosomes	PEG precipitation	Intravenous	From 2×10^6^ cells	1, 3 and 5 days	Y	Corner test, rotarod, tightrope	6 days
Geng *et al*^29^	2019	Transient MCAO, filament	Rat	Rat AMSC-derived miR-126±exosomes	Precipitation	Intravenous	NK	2 hours	N	Foot fault, mNSS	14 days
Han *et al*^30^	2018	ICH, autologous blood	Rat	Rat BMSC-derived exosomes	Precipitation	Intravenous	Derived from 3×10^6^ cells	24 hours	Y	Modified Morris water maze, mNSS, odour recognition	28 days
Huang *et al*^31^	2018	Transient MCAO, filament	Rat	PEDF-modified AMSC-derived exosomes	Precipitation	Intraventricular	100 µg/kg/day	3 days prior to MCAO	Y	N/A	3 days
Jiang *et al*^32^	2018	Permanent MCAO, filament	Rat	Rat AMSC-derived miR-30d-5p±exosomes	Ultracentrifugation	Intravenous	80 µg	0 hour	Y	N/A	3 days
Kalani *et al*^18^	2016	Transient MCAO, filament	Mouse	Mouse ESC-derived curcumin-loaded exosomes	Ultracentrifugation	Intranasal	10 µL total	1 hour (+daily for 7 days)	Y	Belayev neuroscore	7 days
Lee *et al*^33^	2016	Permanent MCAO, filament	Rat	Human MSC-derived microvesicles, primed with normal/ischaemic brain extract	Ultracentrifugation (sucrose cushion), OptiPrep	Intra-articular	0.2 mg/kg	48 hours	Y	Beam balance, modified torso twisting, mNSS, prehensile test	7 days
Liu *et al*^34^	2019	Transient MCAO, filament	Rat	Rat BMSC (transferrin expressing)-derived enkephalin-loaded exosomes	Ultracentrifugation	Intravenous	500 µL × 1×10^5^/mL	2 hours	N	Forelimb flexion, inclined board test, 5-point neuroscore	21 days
Moon *et al*^35^	2019	Transient MCAO, filament	Rat	Rat MSC or fibroblast-derived exosomes	Centrifugation at 14 000 *g*	Intravenous	30 µg	24 hours	N	Cylinder test, ladder walking, mNSS	14 days
Otero-Ortega *et al*^36^	2017	Transient subcortical, endothelin-1	Rat	Rat AMSC-derived exosomes	Precipitation	Intravenous	100 µg	24 hours	Y	Beam walking, modified Rogers test, rotarod	28 days
Otero-Ortega *et al*^37^	2018	ICH, collagenase	Rat	Rat AMSC-derived exosomes	Precipitation	Intravenous	100 µg	24 hours	Y	Beam walking, modified Rogers test, rotarod	28 days
Pan *et al*^38^	2016	Transient MCAO, filament	Mouse	Human brain microvascular cell-derived exosomes	Centrifugation at 20 000 *g*	Intravenous	50 µg	30 min	Y	5-point neuroscore	48 hours
Shan *et al*^19^	2013	Transient MCAO, filament	Rat	Microparticles from reperfusion-ischaemia injury or healthy rats	Ultracentrifugation	Intravenous	NK	30 min prereperfusion	Y	18-point neuroscore	9 days
Webb *et al*^16^	2018	Transient MCAO, thromboembolic	Mouse	Human NSC and MSC-derived EVs	Ultrafiltration	Intravenous	NK	2, 14+38 hours	Y	Adhesive removal, beam walking, foot fault, hanging wire, neurological deficit score, novel object recognition, tail suspension	48 hours
Webb *et al*^17^	2018	Permanent MCAO, electrocoagulation	Pig	Human NSC EVs	Ultrafiltration	Intravenous	2.7×10^10^ vesicles/kg	2, 14+24 hours	Y	Gait analysis, open field	24 hours
Xin *et al*^39^	2013	Transient MCAO, filament	Rat	Rat BMSC-derived exosomes	Ultracentrifugation, sucrose step gradient	Intravenous	100 µg	24 hours	Y	Foot fault, mNSS	28 days
Xin *et al*^40^	2017a	Transient MCAO, filament	Rat	Rat BMSC-derived, wt/MiR-133b-/MiR-133b+exosomes	Ultracentrifugation	Intra-articular	3×10^11^ particles	24 hours	N	Foot fault, mNSS	28 days
Xin *et al*^41^	2017	Transient MCAO, filament	Rat	Rat BMSC-derived MiR-17-92 cluster+exosomes	Ultracentrifugation, sucrose step gradient	Intravenous	100 µg	24 hours	N	Foot fault, mNSS	28 days
Zheng *et al*^15^	2019	Transient MCAO, filament	Rat	Mouse macrophage-derived exosomes (LPS stimulated)	Ultracentrifugation	Intravenous	2 mg	6 and 24 hours	Y	Belayev and Longa neuroscores	24 hours

AMSC, adipose-derived mesenchymal stem cell; BMSC, bone marrow-derived mesenchymal stem cell;ESC, embryonic stem cell; EV, extracellular vesicle; ICH, intracerebral haemorrhage; LPS, lipopolysaccharide; MCAO, middle cerebral artery occlusion; mNSS, modified neurological severity score; MSC, mesenchymal stem cell;N/A, not applicable; NK, not known; NSC, neural stem cell;PEDF, pigment epithelial-derived factor; PEG, polyethylene Glycol; wt, wild type.

The vast majority of studies (n=15) used EVs derived from MSCs (table 1). Other studies used macrophage-derived,^15^ neural stem cell-derived^16 17^ or embryonic stem cell-derived^18^ EVs. Another group isolated EVs from blood of animals having undergone ischaemia/reperfusion injury.^19^ A total of 13 studies (65%) reported data on the characterisation of EVs (table 2). Studies also differed in their methods to isolate EVs, with the majority using ultracentrifugation (n=10) or precipitation (n=6). Other studies used ultrafiltration (n=2) or centrifugation at various speeds (n=2).

**Table 2:** Extracellular vesicle characterisation as reported in included papers

First author	Year	Data shown in paper	Size/morphology		Membrane-associated markers									Cytosolic proteins recovered in EVs			
			Nanoparticle tracking	Microscopy	CD63	CD81	CD9	CD31	CD144	CD45	CD29	CD41b	MCSP	TSG101	Alix	HSP70	Annexin V
Chen 27	2016	x			*									*			
Doeppner 28	2015	x				*								*			
Geng^29^	2019	x															
Han^30^	2018	x		*											*		
Huang^31^	2018	✓		✓ TEM	WB	WB								WB			
Jiang^32^	2018	✓	✓	✓ TEM	WB	WB	WB							WB			
Kalani^18^	2016	✓	✓											WB			
Lee^33^	2016	x															
Liu^34^	2019	✓	✓	✓ cryo-EM	WB									WB		WB	
Moon^35^	2019	✓	✓	✓ TEM													
Otero-Ortega^36^	2017	✓	✓	✓ EM		IF									WB		
Otero-Ortega^37^	2018	✓	✓	✓ EM	IF										WB		
Pan^38^	2016	✓	✓					FC	FC								FC
Shan^19^	2013	✓		✓ cryo-EM					FC	FC							FC
Webb^16^	2018	✓		✓ EM													
Webb^17^	2018	✓	✓	✓ SLIM							FC	FC	FC				
Xin^39^	2013	x															
Xin^40^	2017a	✓		✓ TEM	WB	WB											
Xin^41^	2017b	x		*	*										*		
Zheng^15^	2019	✓	DLS	✓ TEM		WB	WB							WB	WB		

First International Society for Extracellular Vesicles (ISEV) guidelines on EV characterisation and reporting released in 2014, updated in 2018.

*Denotes characterisation reported but not presented.

DLS, dynamic light scattering; EM, electron microscopy; EV, extracellular vesicle; FC, flow cytometry; IF, immunofluorescence; SLIM, spatial light interference microscopy; TEM, transmission electron microscopy; WB, western blot.

### Quantitative synthesis: meta-analysis

**Figure 2:**
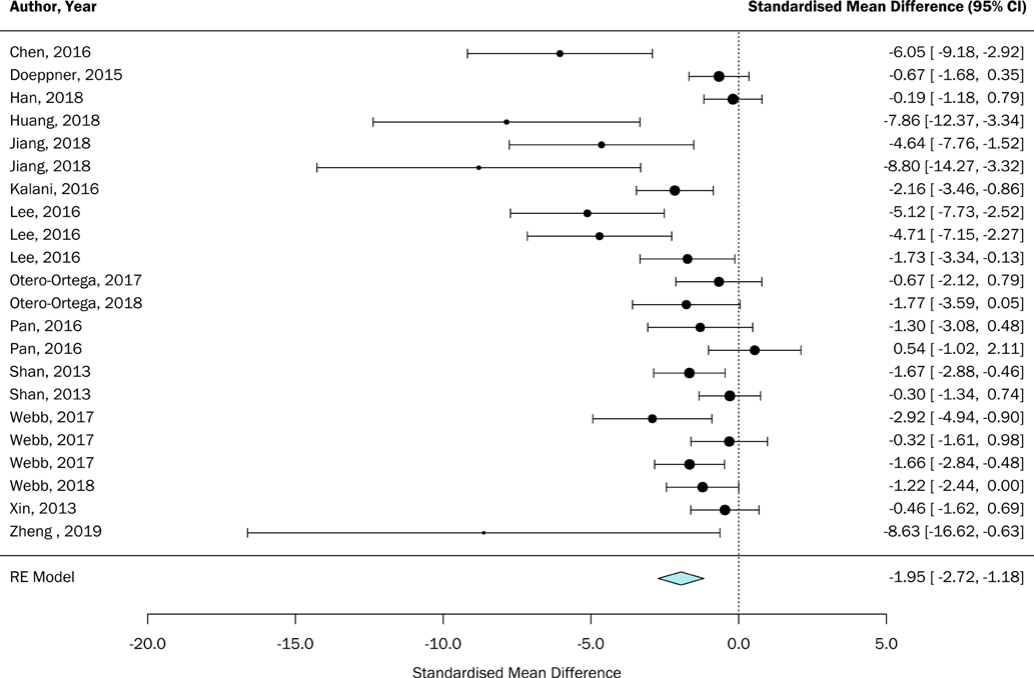
Effects of extracellular vesicle (EV) interventions on lesion volume. Forest plots of standardised mean difference and 95% CI. RE, random effects.

**Figure 3:**
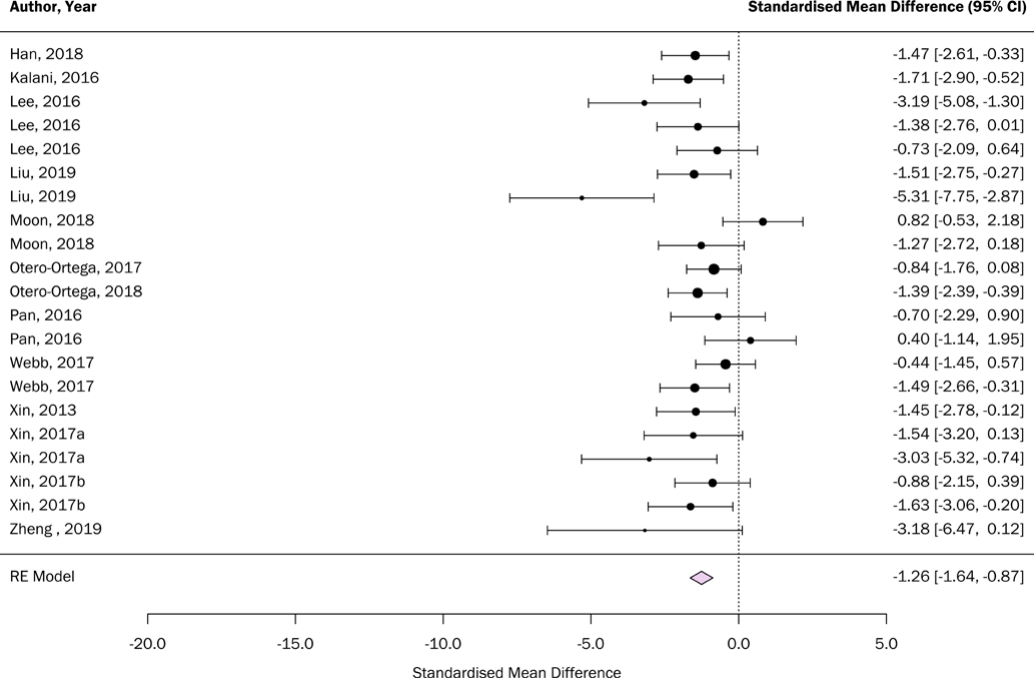
Effects of extracellular vesicle (EV) interventions on neurological score. Forest plots of standardised mean difference and 95% CI. RE, random effects.

Meta-analysis was performed on data extracted for lesion volume and neurological score. Overall, lesion volume was reduced in animals receiving EVs compared with controls (SMD: −1.95, 95% CI −2.72 to 1.18, figure 2). Similarly, neurological scores were improved in EV-treated groups (SMD: −1.26, 95% CI −1.64 to 0.87, figure 3). As can be seen from both figures, interstudy heterogeneity was greater for lesion volume outcome compared with neurological score (I^2^=79.51% and 37.77%, respectively).

Subgroup analysis was performed for lesion volume and neurological score (table 3). Effect sizes in lesion volume were significantly different (p=0.026) in studies that randomised animals to experimental groups (−4.36 for studies without randomisation and −1.34 for studies with randomisation). For lesion volume, effect size for intra-arterial administration was significantly greater than for intravenous EV administration (−3.66 to –1.25, p=0.042). Administration of EVs at 0–23 hours after stroke appeared to be the most effective timepoint for treatment for both outcome measures, with effect sizes of −3.53 for lesion volume and −1.64 for neurological score (table 3).

**Table 3:** Subgroup meta-analysis for lesion volume and neurological score

Factor	SMD (95% CI)	I^2^ (%)	Q statistic P value	(df)	Subgroup analysis P value
**Lesion volume**					
Randomisation					0.026
Yes (n=16)	−1.34 (−1.95 to −0.725)	62.6	39.2 0.0006	15	
No (n=6)	−4.36 (−6.94 to −1.77)	86.4	28.4 <0.0001	5	
Blinding to stroke					0.163
Yes (n=13)	−1.59 (−2.20 to −0.97)	58.35	29.1 0.0038	12	
No (n=9)	−3.31 (−5.65 to −0.97)	91.08	42.6 <0.0001	8	
Blinding to outcome					0.172
Yes (n=17)	−1.65 (−2.39 to −0.91)	74	51.1 <0.0001	16	
No (n=5)	−3.93 (−7.11 to −0.75)	90.7	21.6 0.0002	4	
EV characterisation					0.412
Yes (n=15)	−1.60 (−2.30 to −0.90)	60	39.9 0.0003	14	
No (n=7)	−2.39 (−4.14 to −0.64)	89.2	32.9 <0.0001	6	
Timepoint of administration					Prior vs 0–23 hours: 0.761 Prior vs 23–48 hours: 0.666
Pretreatment (n=3)	−2.74 (−6.72 to 1.24)	94.7	11.7 0.0029	2	Prior vs multiple: 0.509
0–23 hours (n=5)	−3.53 (−6.64 to −0.411)	86.7	25.3 <0.0001	4	0–23 hours vs 23–48 hours: 0.324
23–48 hours (n=7)	−1.82 (−3.16 to −0.478)	80.7	23.4 0.0007	6	0–23 hours vs multiple: 0.188
Multiple (n=7)	−1.39 (−2.05 to −0.720)	34.3	11.5 0.0747	6	23–48 hours vs multiple: 0.569
Route of administration					
Intravenous (n=17)	−1.25 (−1.83 to −0.670)	58.1	43.8 0.0002	16	Intravenous vs intra-arterial: 0.042
Intra-arterial (n=3)	−3.66 (−5.91 to −1.41)	68.2	6.77 0.0339	2	Intravenous vs other: 0.239
Other (n=2)	−4.58 (−10.1 to 0.937)	82.3	5.64 0.0176	1	Intra-arterial vs other: 0.762
**Neurological score**					
Randomisation					0.12
Yes (n=15)	−1.07 (−1.52 to −0.622)	36.6	24.2 0.0433	14	
No (n=6)	−1.64 (−2.20 to −1.08)	0	10.2 0.0705	5	
Blinding to stroke					0.902
Yes (n=10)	−1.28 (−1.68 to −0.874)	4.51	11.1 0.270	9	
No (n=11)	−1.22 (−1.98 to −0.465)	64.2	26.3 0.0034	10	
Blinding to outcome					0.224
Yes (n=18)	−1.12 (−1.49 to −0.747)	25.7	26.0 0.0744	17	
No (n=3)	−2.54 (−4.80 to −0.277)	83.8	8.30 0.0157	2	
EV characterisation					0.49
Yes (n=16)	−1.20 (−1.69 to −0.708)	50	32.3 0.0059	15	
No (n=5)	−1.48 (−2.09 to −0.866)	0	4.35 0.361	4	
Timepoint of administration					
Pretreatment (n=0)	N/A	N/A	N/A	N/A	0–23 hours vs 23–48 hours: 0.715
0–23 hours (n=4)	−1.64 (−3.87 to 0.60)	86.7	15.6 0.0014	3	0–23 hours vs multiple: 0.765
23–48 hours (n=13)	−1.21 (−1.63 to −0.796)	20.1	17.6 0.128	12	23–48 hours vs multiple: 0.894
Multiple (n=4)	−1.27 (−2.08 to −0.466)	31.7	4.46 0.212	3	
Route of administration					
Intravenous (n=15)	−1.10 (−1.55 to −0.650)	42.3	28.9 0.0107	14	Intravenous vs intra-arterial: 0.208
Intra-arterial (n=5)	−1.74 (−2.63 to −0.854)	30.6	5.88 0.209	4	Intravenous vs other: 0.353
Other (n=1)	−1.71 (−2.90 to −0.516)	N/A	N/A	N/A	Intra-arterial vs other: 0.961

EV, extracellular vesicle; N/A, not applicable (for subgroups with fewer than two members where comparisons could not be made); SMD, standardised mean difference.

### Risk of bias and study quality

Risk of bias was assessed using the CAMARADES checklist.^14^ The median score was 7, with an IQR of 5–8 (table 4). All studies analysed were published after publication of the Animal Research: Reporting of In Vivo Experiments guidelines for reporting animal experiments,^20^ though in particular the use of comorbid animals and reporting of sample size calculations to power studies were very low at 5% and 15% respectively.

**Table 4:** Study quality as assessed by CAMARADES risk of bias checklist^14^

	% of included studies
(1) Publication in peer-reviewed journal	100
(2) Statement of control of temperature	75
(3) Randomisation of treatment or control	60
(4) Allocation concealment	45
(5) Blinded assessment of outcome	75
(6) Avoidance of anaesthetic with marked intrinsic properties	90
(7) Use of comorbid animals	5
(8) Sample size calculation	15
(9) Statement of compliance with regulatory requirements	100
(10) Statement regarding possible conflict of interest	90
Median study quality (IQR)	7 (5–8)

**Figure 4:**
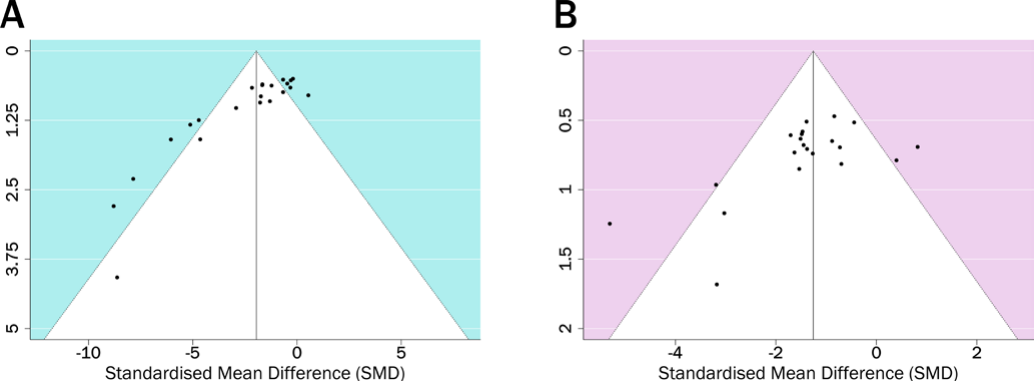
Publication bias assessed by funnel plots for lesion volume (A) and neurological score (B). White funnel area denotes 95% CIs for publication bias.

**Figure 5:**
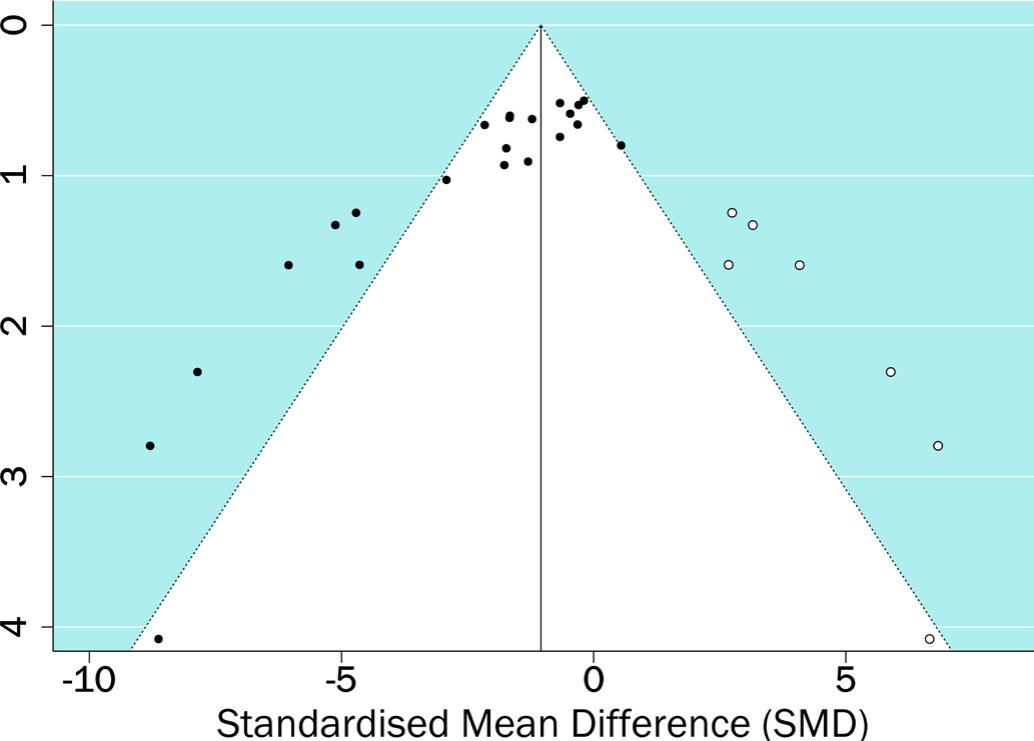
Trim and fill analysis of lesion volume showing published (filled circles) and unpublished (empty circles) studies.

Publication bias was assessed and presented as funnel plots (figure 4). Asymmetry was confirmed for each outcome measure using Egger’s regression test (p<0.0001 for lesion volume, p=0.0054 for neurological score). Trim and fill analysis predicted there are seven unpublished studies with neutral or negative impacts on lesion volume (figure 5) which when accounted reduced the effect size from −1.95 to −1.05. In contrast, no missing studies were estimated for neurological score data.

## Discussion

### Summary of findings

This study aimed to assess the efficacy of EVs in preclinical models of stroke. A total of 20 studies met the inclusion criteria, with a total of 43 comparisons across 19 studies assessed in the meta-analysis. Overall, we found that administration of EVs improved lesion volume and neurological scores. Administration of EVs from 0 to 23 hours seemed the most effective timepoint for treatment, as effect sizes were greater for both outcome measures. Study quality was assessed using the CAMARADES checklist,^14^ finding a median score of 7, though with poor utilisation of comorbid animals (5%) and sample size calculations (15%). Funnel plots were asymmetrical as confirmed by Egger’s regression test, suggesting there was publication bias. Trim and fill analysis identified there are seven unpublished studies reporting neutral or negative lesion volume data but none for neurological score.

### Heterogeneity in therapeutic EV studies

While most of the studies included in this review used MSCs for EV production, several other cell types were used such as neural stem cells and macrophages. The composition of EVs is largely dependent on the cell of origin and culture conditions,^21^ which may explain much of the heterogeneity in these studies. EV technology is still relatively new, and as such there is no ‘gold standard’ method for EV isolation. The 2018 ISEV guidelines^22^ suggest methods should be selected based on the recovery and specificity required for downstream applications. The more common methods (ultracentrifugation and precipitation) are suggested to have low to intermediate specificity for EV subtypes, meaning that other non-vesicular components or mixed EV populations may have been coisolated and affected the final therapeutic EV composition. The differences in the techniques used across the studies included in this review are therefore another potential source of heterogeneity affecting outcome measures. Putting aside diverse EV compositions, it is also important to consider the treatment regimens employed in the included studies, in which EVs were injected via different routes, in different stroke models, at different timepoints relative to stroke onset and at different doses. As the pathophysiological response to stroke is highly dynamic and involves recruitment of different cells in the acute and chronic stages, it is unsurprising that the timepoint of EV administration appeared to affect the lesion volume and neurological scores.

### Issues with outcome measures

A significant issue for the stroke field is the lack of standardised testing for functional deficits. Hietamies and colleagues^23^ identified 74 functional outcome measures reported across 636 preclinical stroke studies, the majority of which were neurological deficit scores of varying scales. Although useful to compare data across multiple studies, the differing scales and criteria used to detect deficits can confound studies. Furthermore, due to the nature of scoring, neurological deficit scores are subjective and can give false positive scores.^24^ Rodents show spontaneous recovery in weeks following stroke onset, which may render neurological scores ineffective at later timepoints due to their insensitivity to more complex deficits.^24^ Other behavioural tests that may be more appropriate for detecting long-term functional deficits include staircase test catwalk analysis.^24 25^ Finally, Menezes *et al*^26^ suggest that lesion volume may be less relevant than lesion location and topology with regard to stroke severity, which raises the question of whether lesion volume alone should be used as an outcome measure for preclinical stroke studies.

### Limitations

It is important to note that data were extracted from the final timepoints of each study in order to observe the longer term effects of the EV treatments. However, studies were not conducted for the same number of days after stroke onset, with the shortest sacrificing animals at 24 hours, and the longest at 60 days. As mentioned above, spontaneous recovery occurs after stroke onset, and thus effect sizes for neurological scores may be confounded by the timepoints; the shortest study by Zheng *et al*^15^ had the third highest calculated effect size, though longer studies may have had larger effect sizes, had the data been extracted from more acute timepoints.

Our study was limited by the articles included; several studies did not measure either lesion volume or neurological score. This is unsurprising given the number of functional outcome measures characterised, though it means potentially relevant data regarding EV efficacy in preclinical stroke models were not included in this review. The decision to use these outcome measures was made based on the number of studies employing these measures, to capture the maximum number of relevant studies that could be easily compared.

While extracting study design data, we noticed substantial heterogeneity across the included studies, with regard to timepoint and routes of EV administration. For this reason, we assigned studies to subgroups we believed may be relevant to the reported outcomes. However, due to the paucity of studies, some subgroups were small and disproportionate, meaning that subgroup analysis is unlikely to have been sufficiently powered. Indeed, only one comparison for randomisation for lesion volume was statistically significant. We did not account for heterogeneity arising from usage of EVs from different sources and with various modifications, as the EVs and their contents have not been fully characterised, making subgroups challenging to define.

## Conclusions

Overall, EV-based interventions had positive impacts on lesion volume and neurological score in preclinical stroke models, indicating that EVs may hold great potential for future translational stroke research. Subgroup analyses may have been confounded by considerable heterogeneity in study design and the low number of included studies. However, given their efficacy across studies with notably different treatment regimens, the positive effects of EVs may be wide ranging in the contexts of neuroprotection, repair and regeneration. To fully unlock the potential of EVs as therapy for stroke, it is important to determine the mechanisms by which they elicit their effects, perhaps investigating the roles of specific cargo contained in the EVs. Translation of preclinical EV research into a clinical setting will likely require methodological standardisation and improvements in EV characterisation in order to meet regulatory requirements for human therapy. Interest in therapeutic EVs is expanding, with study numbers increasing yearly, therefore we expect that a clearer understanding of the most effective EVs and treatment regimens may soon come to light.
